# Mapping research trends of uveal melanoma: a bibliometric analysis

**DOI:** 10.1007/s10792-021-02098-0

**Published:** 2021-11-05

**Authors:** Senmao Li, Yongwei Guo, Xiaoyi Hou, Jinhua Liu, Wanlin Fan, Sitong Ju, Philomena A. Wawer Matos, Alexander C. Rokohl, Ludwig M. Heindl

**Affiliations:** 1 Department of Ophthalmology, Faculty of Medicine, University Hospital Cologne, University of Cologne, Kerpener Strasse 62, 50937 Cologne, Germany; 2 Eye Center, Second Affiliated Hospital, School of Medicine, Zhejiang University, Hangzhou, Zhejiang China; 3 Center for Integrated Oncology (CIO), Aachen-Bonn-Cologne-Duesseldorf, Cologne, Germany

**Keywords:** Uveal melanoma, Bibliometric analysis, Knowledge domain, Visualization, VOSviewer

## Abstract

**Background:**

To explore the research trends for uveal melanoma with bibliometric methods using Web of Science Core Collection (WoSCC) and PubMed (PM).

**Methods:**

To find UM-related studies, “uveal melanoma” was used as search term in the WoSCC and PM for the period time from 2000 to 2020. Bibliographic coupling analysis was used to investigate the journals with the highest number of UM-related publications. VOSviewer (VV) was used for mapping the knowledge domain and visualizing the co-occurrence of terms, authors, organizations, countries, co-citation literature, and keywords. The knowledge map based on WoSCC and PM was compared.

**Results:**

In the WoSCC 3,748 articles were found, while in PM the search resulted in 3,403 articles. The number of original articles has steadily grown in general in the past two decades. The top ten authors were contributing to 23% (*n* = 856) of all publications, while the top 10 institutions published 41% (*n* = 1524) of all articles. The top 3 journals with the highest number of publications for UM-related research included *Investigative ophthalmology & visual science*, *Ophthalmology,* and *British Journal of Ophthalmology*. Co-occurrence analysis based on author keywords showed 6 clusters*.* The most frequent keywords included are metastasis, prognosis, and brachytherapy. The latest research hotspots focused on *BAP1*, immunotherapy and *GNAQ.*

**Conclusions:**

Genetics and immunology are the latest research frontiers in uveal melanoma. There is a clear need for interdisciplinary, molecular and clinical research approaches to improve the fatal prognosis of uveal melanoma patients.

## Background

Uveal melanoma (UM) is a common primary intraocular malignancy in Caucasians [1]. 85% of ocular melanomas are UM [2]. Unfortunately, this common malignant tumor has a poor prognosis and low survival rate. Metastasis to the liver is common, and half of posterior UM patients suffered from this within 15 years after the initial diagnosis and treatment [3]. Meanwhile, the 5-year all-cause mortality rate following enucleation for choroidal melanoma ranges from 16 to 53% [1]. Compared to common ophthalmic diseases, the number of studies and research on UM is limited. However, some research papers have been published in the last 20 years. Despite the relatively limited number of articles regarding UM research, researchers cannot read all of the UM literature, and abstracts, as a traditional type of literature, do not provide us with information on hot topics and current trends in the field of UM research.

Web of Science Core Collection (WoSCC) and PubMed (PM) provide established databases for literature search and bibliometric analysis, a method using mathematical statistics to analyze relevant literature, is now firmly established as scientific specialties and is an integral part of research evaluation methodology, especially within the scientific and applied fields [4]. With bibliometric analyses, it is possible to assess the growth in publications, international collaborations, author productivity, or keyword co-occurrence related to UM studies. Using visualization tools, a knowledge domain map through bibliometric hotspots and connections can be generated.

This study aims to explore the current research status of uveal melanoma related studies by mapping the knowledge domain with bibliometric methods using Web of Science Core Collection and PubMed, to identify research trends and to provide researchers a better overview and more information about UM related research over the last 20 years.

### Methods

The Science Citation Index Expanded database in the Web of Science Core Collection (WoSCC) was included in this study. The searching keyword is Uveal melanoma, the document type was article, and the period was "from 2000 to 2020." No language restrictions were set. The database sends back 3748 matched articles.

The MEDLINE database of references and abstracts on life sciences and biomedical topics with PubMed access was retrieved online as the study source. The searching keyword is Uveal melanoma, and the search resulted in 3403 articles from 2000 to 2020. The query is "uveal melanoma"[Supplementary Concept] OR "uveal melanoma"[All Fields] OR "uveal melanoma"[All Fields] Filters: from 2000 to 2020. Data cleaning, identifying inaccurate or irrelevant parts of the data, and then replacing them were done manually.

Excel (2016; Microsoft) was used to create a database based on WoSCC and PubMed-generated data. VOSviewer [5] (1.6.15; Leiden University) was used to mapping the knowledge domain and visualizing the co-occurrence of terms, authors, organizations, countries, co-citation literature, and keywords.

For analysis modes, the bibliographic coupling analysis was performed for source journals and co-occurrence analysis for keywords. In Bibliographic coupling analysis, the relatedness of items is determined based on the number of references they share. In co-occurrence analysis, the relatedness of items is determined based on the number of documents they occur together.

For the knowledge map, the node size defines the number of published articles. The links between nodes represent relatedness. The color of a node determines the cluster it belongs to, and different clusters are represented by different colors.

### Results

#### Annual publications distribution

**Fig. 1:**
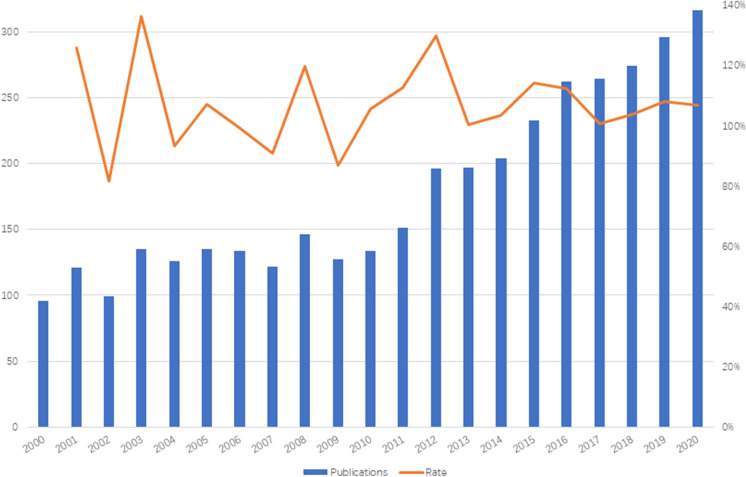
Annual publications distribution and growth rate. Growth rate = 1 + (this year / last year) × 100%

Based on WOSCC, the publication distribution was shown in Fig. 1. The number of average annual publications is 179.43 ± 68.90, and the average annual growth rate is 7.05%. The number of publications has steadily grown in general in the past two decades. The literature growth rate has been positive for the past 10 years, with peaks of growth in 2003, 2012, and 2015. The growth rate of 30% in 2013 was the highest within the last 20 years.

### Research institutions and country and authors distribution

There are 2,650 institutions that published 3,748 articles. The top 10 institutions published 40.66% of all publications, which is 1,524 articles. The rank was organized in Table 1. In the last two decades, 3,768 matched articles originated from 78 countries. The top 5 countries included USA, Germany, UK, China, and the Netherlands. Due to the imbalanced institution distribution, co-authorship analysis was not performed. Authors and research institutions are highly correlated, and the distribution of research institutions is closely linked to the distribution of countries. A total of 13,979 authors contributed research to this area, with the top ten contributing 22.84% (n = 856) of all literature seen in Table 2.

**Table 1:** The top 10 most productive institutions of UM researching in last two decades

Institutes	Papers	%
Jefferson University	201	5.334
University of California System	199	5.281
Leiden University	178	4.724
Unicancer	161	4.273
University of Liverpool	151	4.007
Harvard University	149	3.954
University of Texas System	139	3.689
University of Duisburg Essen	119	3.158
Royal Liverpool Broadgreen University Hospitals NHS Trust	114	3.025
Royal Liverpool University Hospital	113	2.999

**Table 2:** The top 10 most productive authors contributed of UM researching in last two decades

Name	Papers	%
Shields CL	148	3.949
Jager MJ	119	3.175
Shields JA	117	3.122
Coupland SE	89	2.375
Harbour JW	74	1.974
Damato B	71	1.894
Luyten GPM	67	1.788
Singh AD	62	1.645
Desjardins I	56	1.494
Burnier MN	53	1.414

### Source journals analysis

**Fig. 2:**
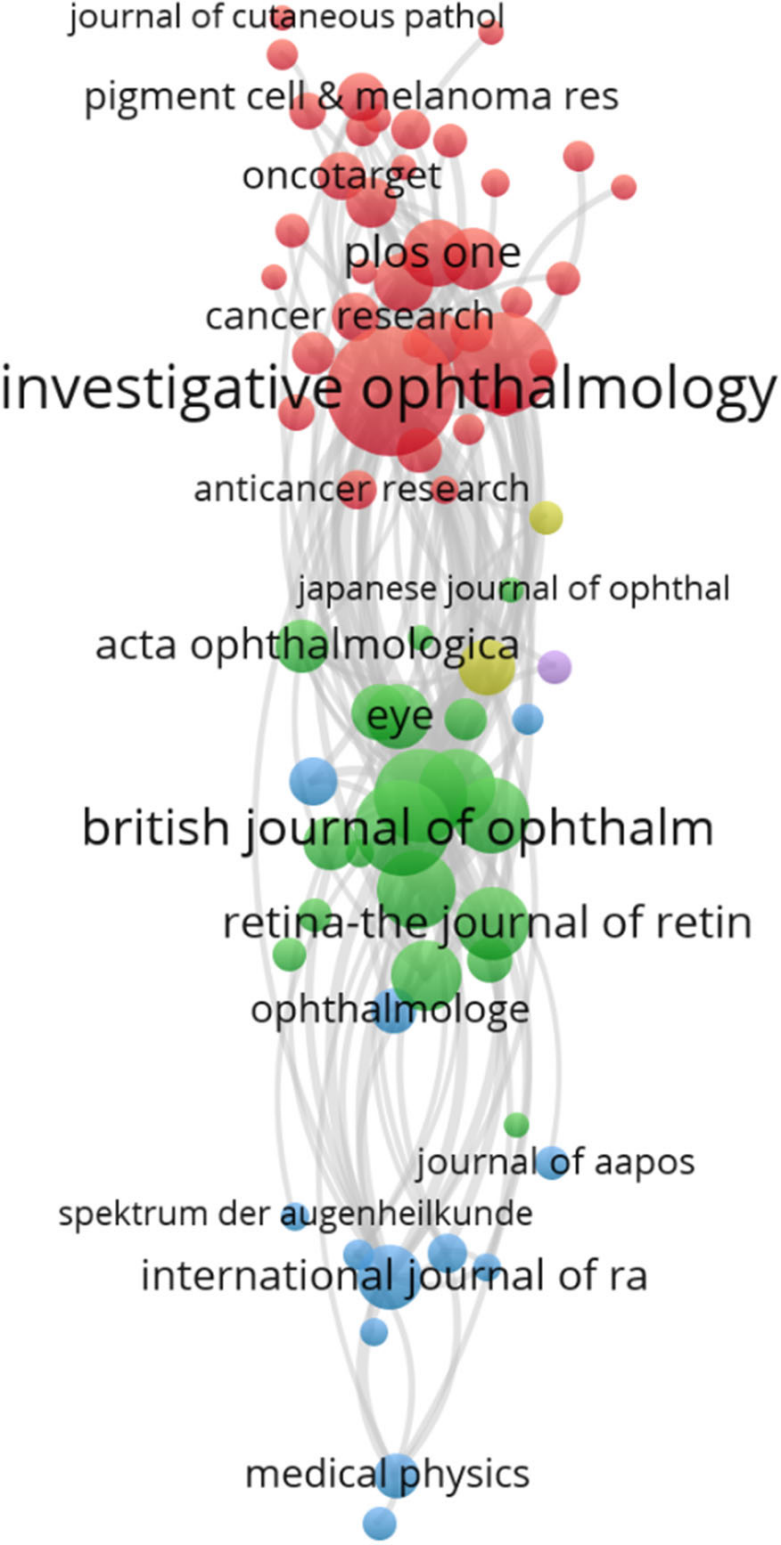
The bibliographic coupling analysis visualization of sources. The node size is determined by the number of published articles. In Bibliographic coupling analysis, the relatedness of items is determined based on the number of references they share. The links between nodes represent relatedness. The color of a node represents the cluster it belongs to, and different clusters are represented by different colors

Based on WoSCC, the bibliographic coupling analysis of source journals was performed. The top 10 journals were organized in Table 3. *Investigative ophthalmology & visual science* accepted most papers about UM (223, 5.95%); *Ophthalmology* accepted 114 articles (3.04%); *British Journal of Ophthalmology* accepted 126 (3.36%). The top 3 journals accepted 12.35% of articles in the past two decades. The bibliographic coupling analysis visualization is shown in Fig. 2. It is worth noting that in the distribution of journals, it is clear that there are 3 main blocks. The clusters are very close together, but the connections between different clusters are very far apart. In the knowledge map, greater distances indicate fewer connections. This distribution indicates that the research hotspots covered by individual journals are concentrated and limited, and it is not easy to find suitable journals for publication of cross-disciplinary research. The top three journals in the ranking are located in the first and second largest clusters, with no representative journals appearing in the third cluster. Additionally, according to total link strength, although *Investigative ophthalmology & visual science* includes more literature, *Ophthalmology* has a higher total link strength, meaning that it publishes articles that are more recognized by researchers as more influential.

**Table 3:** The top 10 most valuable sources of UM researching in last two decades

Sources	Documents	Citations	Total link strength
Investigative Ophthalmology and Visual Science	223	5440	2667
Ophthalmology	114	4778	2488
British Journal of Ophthalmology	126	1881	1359
Archives of Ophthalmology	77	2404	1159
American Journal of Ophthalmology	81	1289	1082
Melanoma Research	135	2034	1020
Eye	58	1104	858
International Journal of Radiation Oncology Biology Physics	59	1568	778
Clinical Cancer Research	47	1832	735
Jama Ophthalmology	45	724	734

The relatedness of items is determined based on the number of references they share. Total link strength attribute indicates the total strength of the bibliographic coupling links of a given sources with other sources

### Keywords distribution and analysis

**Fig. 3:**
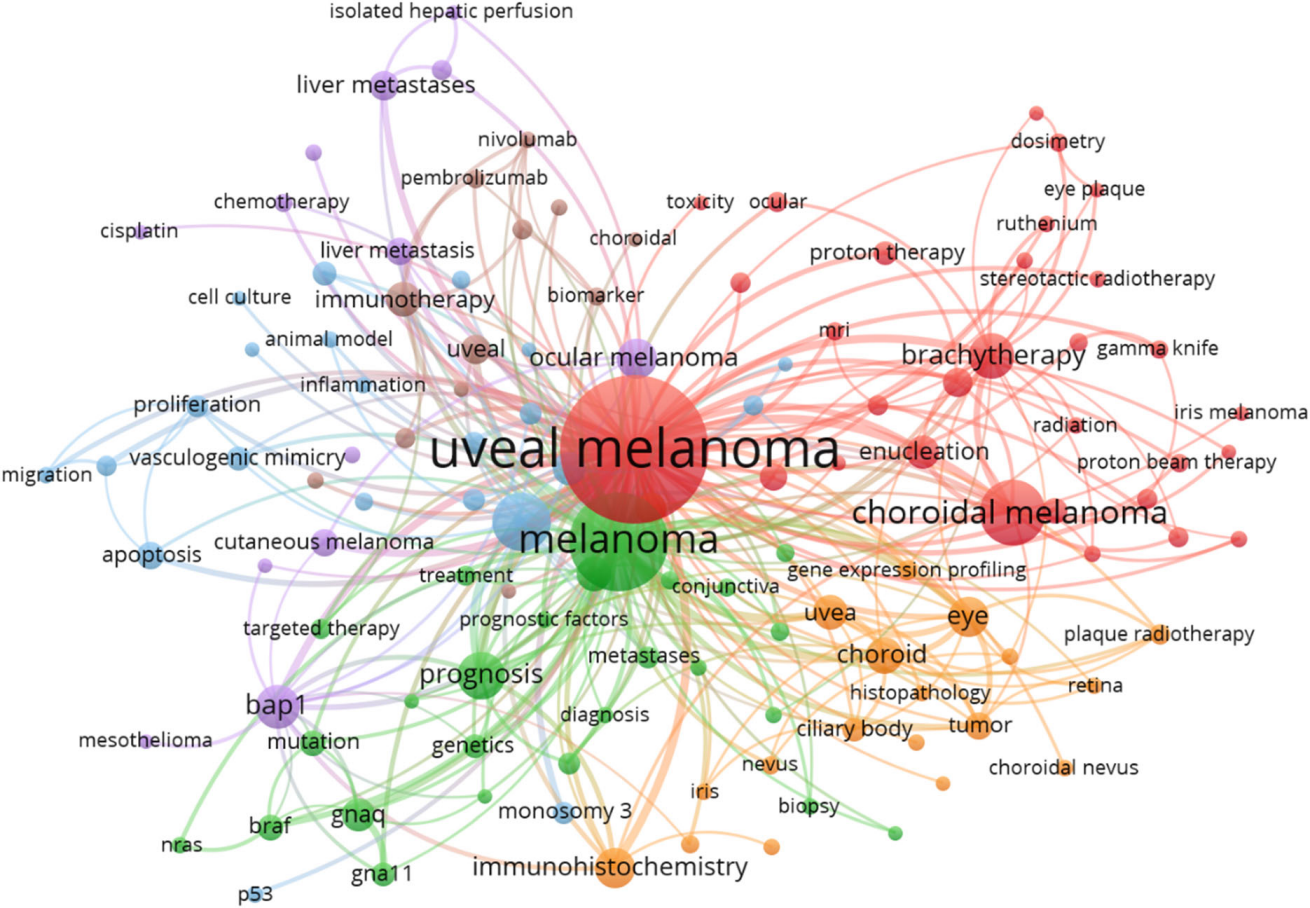
The co-occurrence analysis visualization of keywords in WoSCC. The node size is determined by the number of published articles. In co-occurrence analysis, the relatedness of items is determined based on the number of documents they occur together. The links between nodes represent relatedness. The color of a node represents the cluster it belongs to, and different clusters are represented by different colors

According to co-occurrence analysis based on author keywords of WoSCC, the hotspots of keywords related to UM were found. We set a minimum number of occurrences of a keyword as 10. Of the 4286 keywords, 121 meet the threshold. Similar keywords will be clustered together, eventually forming six major clusters, marked in 6 colors (Fig. 3). By filtering or combining similar keywords, the following keywords were found to be more influential according to the total link strength: metastasis (308), prognosis (221), brachytherapy (192), *BAP1* (154), immunotherapy (136), immunohistochemistry (134), survival (131), *GNAQ* (102), enucleation (93) and radiotherapy (88). The top 10 keywords in every cluster were listed in Table.4.

**Table 4:** The top 10 keywords in every cluster

1 Red		2 Green		3 Blue		4 Orange		5 Purple		6 Brown	
Uveal melanoma	1256	Melanoma	738	Metastasis	308	Eye	196	*BAP1*	154	Immunotherapy	136
Choroidal melanoma	261	Prognosis	221	Cancer	81	Choroid	168	Ocular melanoma	102	Uveal	93
Brachytherapy	192	Survival	131	Monosomy 3	49	Uvea	154	Liver metastases	66	Ipilimumab	51
Enucleation	93	*GNAQ*	102	Oncology	49	Immunohistochemistry	134	Cutaneous melanoma	63	Mucosal melanoma	45
Radiotherapy	88	Genetics	74	Liver	48	Tumor	108	Liver metastasis	55	Pembrolizumab	42
Retinoblastoma	62	*GNA11*	72	Apoptosis	45	Malignant melanoma	72	Melphalan	44	Nivolumab	41
Ocular	52	Pathology	64	Invasion	43	Ciliary body	54	Chemotherapy	37	Metastatic	31
Proton therapy	46	Mutation	63	Angiogenesis	41	Plaque radiotherapy	48	Isolated hepatic perfusion	26	Biomarker	30
Ultrasound	45	*BRAF*	61	Proliferation	39	Nevus	45	Radioembolization	19	Epigenetics	28
Plaque brachytherapy	44	Treatment	56	Conjunctival melanoma	35	Melanocytoma	44	Breast cancer	18	Metastatic melanoma	24

The more frequent of keyword according to the co-occurrence analysis, the higher rank it gets. The numbers after keywords show the total link strength of keyword according to the co-occurrence analysis

**Fig. 4:**
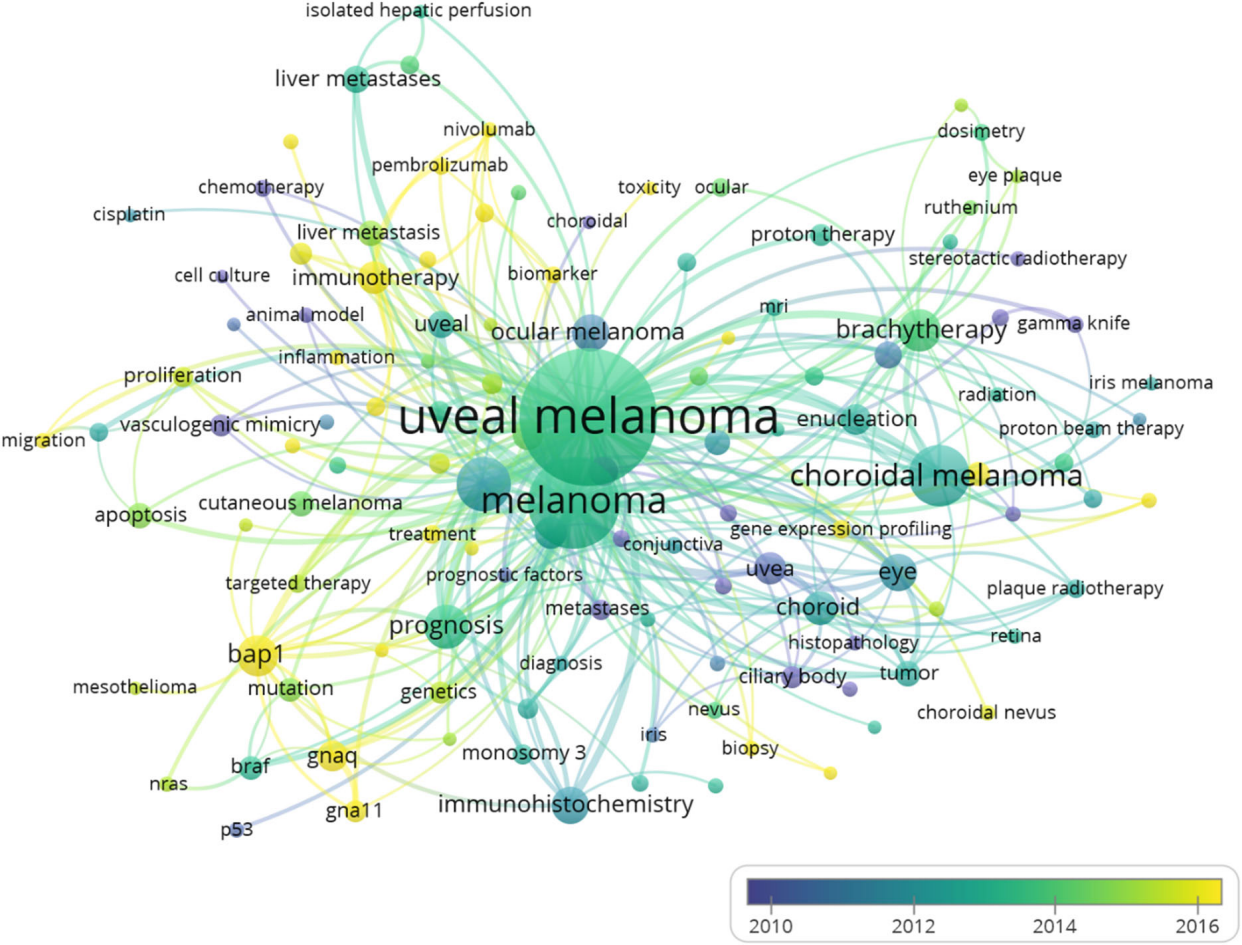
Overly visualization of the co-occurrence analysis visualization of keywords in WoSCC. The node size is determined by the number of published articles. The color of the spots represents mean published time. The bluer the color, the earlier the average published time of the keyword. The yellower the color, the newer the keyword is relatively

By analyzing the average year of first occurrence, among the research hotspots, the following are the highlights of research in recent 5 years: *BAP1* (154), immunotherapy (136), *GNAQ* (102)*, GNA11* (72) and ipilimumab (51). They are mainly concentrated in green, purple and brown clusters. Overly visualization was shown in Fig. 4. Through the images, we can see that genetics and immunology are currently at the frontier of research and recognized by most researchers.

**Fig. 5:**
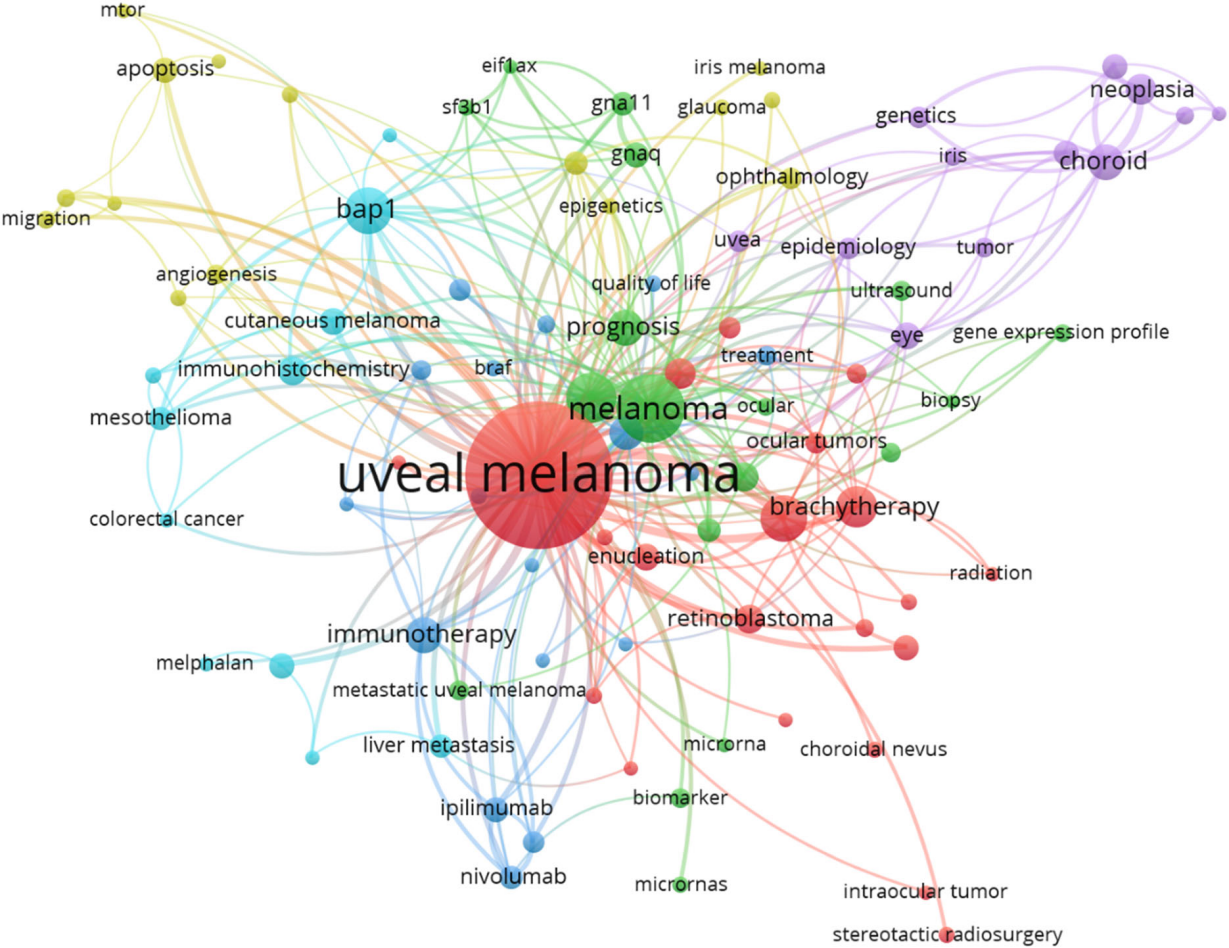
The co-occurrence analysis visualization of keywords in PubMed. The node size is determined by the number of published articles. In co-occurrence analysis, the relatedness of items is determined based on the number of documents they occur together. The links between nodes represent relatedness. The color of a node represents the cluster it belongs to, and different clusters are represented by different colors

According to co-occurrence analysis based on author keywords of PubMed, we set a minimum number of occurrences of a keyword as 5. Of the 2501 keywords, 112 met the threshold. Similar keywords were clustered together, forming six major clusters, marked in 6 colors (Fig. 5). By filtering or combining similar keywords, the following keywords were found to be more influential according to the total link strength: metastasis (99), *BAP1* (74), prognosis (59), immunotherapy (56) and brachytherapy (50). Due to the different formats of these two databases, even though they cover mostly the same literature, the knowledge maps they constitute are different. The knowledge maps constructed from these two databases showed that the hotspots were similar in UM studies.

### Discussion

The development trend of research about uveal melanoma was shown by the quantity variation of academic papers. By analyzing the literature over the last 20 years, we found a steady growth of studies on UM in the last 5 years. As a malignancy, such growth rates reflect a more comprehensive understanding of the disease. And it is noteworthy that there are several time points when the discovery of new related genes might lead to a new wave of research. Although the mechanism of the disease is not fully understood, we do have a better understanding of the disease.

The most productive institutes are mainly located in the United States, the United Kingdom, the Netherlands, France, and Germany. Six of the top 10 research institutions are from Europe. Considering the high-risk population for UM, it is not difficult to infer that Europe and the United States are UM research centers. We are very grateful to these core authors for their contributions to the field, but the significance of the visual analysis results about the authors is not very obvious. These authors are studying this field for many years, so their findings have a longer exposure time. However, the authors' results can be used as a reference indicator when researchers choose their collaborators.

Through the bibliographic coupling analysis of source journals, we can determine the core journals in studies of UM. In Fig. 2, the apparent clustering of journals is determined. The results show a clear stratification of clinical and basic research and uneven distribution. This implicates the difficulties that basic research encounters in translating results into clinical applications. In the top 3 journals, *Ophthalmology* and *British Journal of Ophthalmology* belong to the same cluster, while *Investigative ophthalmology & visual science* belongs to the first cluster. Articles published in these journals are more likely to be cited. In other words, articles in these journals are more likely to disseminate their findings.

For the analysis of keywords, we used co-occurrence analysis. Through this analysis, we can see the hotspots that authors in this field are most concerned about and draw a knowledge background map. Therefore, we conducted a cluster analysis to explore the main topics of UM research. UM, keywords formed six main clusters and clustered together keywords with similar research topics (Fig. 3). Combining the characteristics of UM and the current status of UM research, the 6 clusters are analyzed as follows:

Cluster 1 (Red): in this cluster, keywords are mainly related to traditional therapies. When treating uveal melanoma, the most important thing is to reduce mortality. However, patients and ophthalmologists are also committed to maintaining the visual function, cosmetic appearance, and quality of living [6, 7]. Treating small to medium melanomas with Ru^106^ was a success [8–10]. According to different studies, regular treatments like enucleation, brachytherapy, charged particle irradiation, and local resection have similar survival outcomes [11–13]. It is noteworthy that studies on traditional treatment methods still accounted for the majority, but the studies were generally older.

Cluster 2 (Green): genetically related prognosis of melanoma was focused in this cluster. *GNAQ*, a stimulatory α_q_ subunit of heterotrimeric G-proteins, was found to be mutated in 40% of Ums [14]. Some studies showed that 83% of UM had somatic mutations in *GNAQ* or *GNA11*. Constitutive activation of the pathway involving these two genes appears to be a major contributor to the development of uveal melanoma [15]. These two genes caused a wave of research around 2010. Prior to this, around 2003, research in this area was focused on *BRAF* mutations. Although Mutations in the *BRAF* gene enhance the kinase activity have been described in > 60% of cutaneous melanomas and premalignant melanocytic lesions, it is not common in primary uveal melanoma [16, 17]. Within this cluster, several genetic research hotspots are beginning to emerge, they are strongly clustered in time, and the research is generally close to us.

Cluster 3 (Blue): metastasis of UM is most mentioned in this cluster. In this cluster, the research approach is more oriented toward oncology. Of greatest concern were UM metastasis, apoptosis, and invasion. Monosomy 3 as a significant predictor of both relapse-free and overall survival of UM [18] is in this cluster's leading position. In this cluster, however, there is no trend regarding specific genes or key nodes. This indicates that there are still many unclear mechanisms waiting to be studied in this area. Researchers can look for new research directions in this cluster.

Cluster 4 (Orange): comparing with cluster 3, the research approach is more oriented toward ophthalmology. Within this cluster, the hotspots of research are the choroid, uvea, ciliary body. Immunohistochemistry, as a critical histopathological examination [19], appeals in this cluster. Also appearing is plaque radiotherapy, a common treatment method [20].

Cluster 5 (Purple): most keywords in this cluster mutation of *BAP1* were noticed. Some specific genes such as *BAP1*, *EIF1AX*, and *SF3B1* are related to uveal melanoma metastasis [21] and have prognostic value in UM. Many studies found loss of *BAP1* in uveal melanoma metastasis may be mainly involved in the progression of uveal melanoma to an aggressive, metastatic phenotype [19, 21]. A high prevalence of liver metastases, as a character of UM, is clustered here. Unlike clusters 2 and 3, there is an exact object of study, and the direction of study is essentially the same. Research on this gene is worth continuing and is expected to translate into clinical results.

Cluster 6 (Brown): Since immunotherapy has dramatically changed the treatment approach to cutaneous melanoma [22], it is no surprise that immunotherapy has become the hotspot in the latest cluster. The drugs currently in the spotlight are ipilimumab, pembrolizumab, and nivolumab [23–25]. The biomarker, which is closely related to immunotherapy, appears in this cluster at the same time. In this cluster, the literature's average publication date is relatively new, and the study objects are relatively specific.

By comparing the WoSCC and PubMed databases, PubMed has fewer metadata to analyze, the aggregation effect is not apparent, and the connection is not strong enough to reveal the deep connection between individual studies in the field. This study's limitation is that the PubMed database tags are not uniform, resulting in lower quality of metadata acquisition compared to WoSCC. Therefore, the amount of literature covered in this study is not comprehensive.

In conclusion, the analysis of the UM literature for the past 20 years by using scientific data visualization tools permits researchers to find references for their potential research directions, to determine potential research collaborators, and to find proper journals for publishing their articles regarding UM. More importantly, it identifies current hotspots including immunotherapy, *BAP1*, and *GNAQ,* in UM research for the last two decades.

Since medical research's speed now exceeds the learning speed of general physicians and researchers, it is more important to use scientific tools to organize the unknown knowledge. Databases for standardized management of literature tags are more critical in the knowledge explosion era than knowledge itself. In addition, these results help clinicians to find the newest relevant literature and clinical information for state-of-the-art treatment concepts for UM.

## Data Availability

Not applicable.

## Abbreviations

UM: Uveal melanoma
WoSCC: Web of science core collection
PM: PubMed
